# Single-Base Detection of DNA with Simplified Steps on InGaN Quantum Wells

**DOI:** 10.1021/acs.jpcb.5c00200

**Published:** 2025-04-29

**Authors:** Thi Anh Nguyet Nguyen, Ching-Lung Luo, Fan-Ching Chien, Kun-Yu Lai

**Affiliations:** Department of Optics and Photonics, National Central University, Chung-Li, Taoyuan 32001, Taiwan

## Abstract

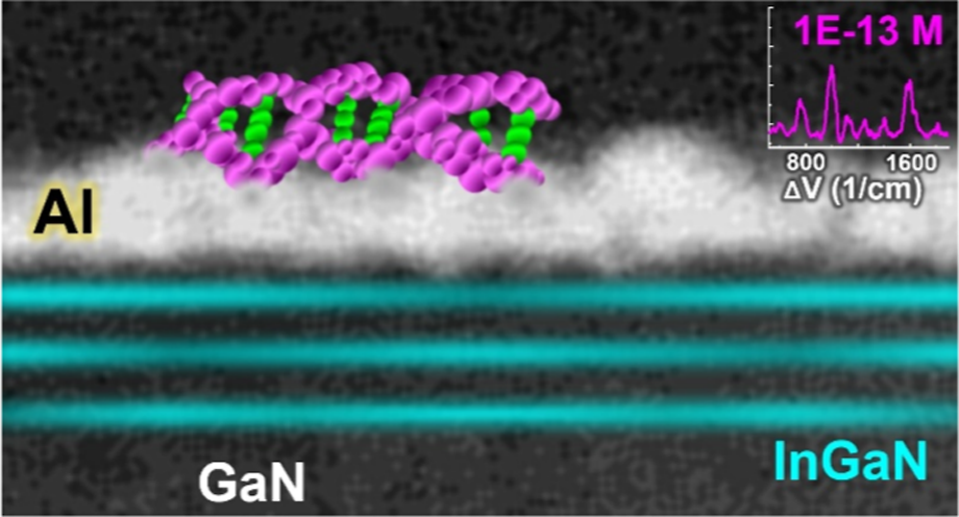

DNA testing is a powerful tool to evaluate an individual’s risk for genetic disorders or certain illnesses. Completing the test quickly and accurately is the key to preventing many deadly diseases. However, the work is usually a tedious process as it entails multiple steps of molecular modification. To address the challenge, we present a simplified DNA detection tactic, skipping surface functionalization, fluorescent labeling, and probe immobilization. In addition, we show that a wide-field (9 × 9 μm^2^) submicron image of dilute DNA (1 × 10^–9^ M) can be captured in a 3.5 min single laser exposure. The task is accomplished by surface-enhanced Raman spectroscopy (SERS) built with InGaN quantum wells covered by Al nanospheres. This unique biochip makes the nucleotide fully exposed to the SERS hot surface, catching the single-base DNA signal with single-molecule sensitivity.

## Introduction

Deoxyribonucleic acid (DNA) testing is becoming an important step in medical procedures. This is because identifying mutations in genes, chromosomes, or proteins of the patients is an effective way to find the right treatment. However, detecting DNA is a demanding task^1−4^ as it usually involves four major steps, i.e., surface functionalization, probe immobilization, fluorescent labeling, and probe–target hybridization. Each step requires multiple hours to complete the binding between functional molecules.^2,3^ The long process not only delays the real-time monitoring but also introduces disturbance signals during the complicated binding procedures.^2−4^ In practice, capturing the mutation is even trickier considering the fact that mutant DNAs can be as few as less than 1% of the wild-type DNA in blood.^1,5^ In other words, simplifying the assay steps without losing the sensitivity is a major hurdle before DNA tests can be further applied to more clinical diagnoses.

Surface-enhanced Raman spectroscopy (SERS) can overcome the hurdle. The technique is empowered by a collective electron oscillation, in resonance with the light inelastically scattered at nanostructured metal/molecule interfaces.^6^ This resonance leads to a million-fold enhancement of the Raman signal from specific molecular vibrations, enabling label-free detection at the single-molecule level.^6−8^ The exceptional sensitivity and selectivity of SERS-based biosensors has galvanized intense research efforts in cancer diagnosis.^1,6^ Like DNA detection, SERS is still not widely available in routine clinical practice, mainly due to the unsatisfactory reliability. This is because the million-fold signal boost of SERS hinges on the surface density of the tiny (<10 nm) hot spot, where the resonant electron oscillation takes place. Since the hot spots on a SERS surface only cover a limited area, it is typically found that less than 1% of the diluted analytes can render detectable signals.^7,8^ For large molecules or long DNA, the few hot spots can only reveal partial Raman fingerprints from specific fragments, hence producing inconsistent spectra from the same analyte.^6^ This spatial limitation becomes even more challenging if we factor in the temporal instability of SERS, often reported as “spectral blinking”.^9^ The problem comes from the thermal diffusion of target molecules across a hot spot upon laser heating,^9^ yielding a sudden burst of SERS intensity up to 1000-fold.^9^ To detect the scarce mutant DNA with a sequence very similar to that of the wild type, acquiring distinct and reliable SERS signals is essentially impossible, let alone achieving it in a rapid manner.

In this work, we demonstrate a label-free thiol-free SERS sensing of DNA with single-base selectivity (detection limit: 2.1 × 10^–12^ M). The analyte adopted here is a 19-mer oligonucleotide with the sequence of the circulating tumor DNA (ctDNA) derived from Kirsten rat sarcoma-2 virus (KRAS) mutation, which is closely tied to pancreatic cancer.^10,11^ This high-throughput, high-performance SERS detection was made possible by InGaN quantum wells (QWs) decorated with Al nanospheres. The excellent carrier trapping of QWs forms a SERS hot surface by significantly densifying the hot spots,^12−14^ and the native surface oxide of Al allows a thiol-free process by providing a natural binding site for the phosphate backbone of nucleotides.^15^ In addition to the simplified single-base detection, we further designed a beam-expansion optical setup, creating a series of Hadamard patterns, to display the submicrometer-resolution (276 × 276 nm^2^) wide-field (9 × 9 μm^2^) SERS imaging of DNA (1 × 10^–9^ M), which was achieved in a short recording time of 3.5 min. All of these results are to make the DNA assay easier and faster, without sacrificing its performance.

## Experimental Section

### DNA Fragment Preparation

19-mer polynucleotides of adenine (A_19_), cytosine (C_19_), thymine (T_19_), guanine (G_19_), probe DNA, target DNA, and wild-type DNA were obtained from Mission Biotech Co., Ltd. (Taiwan). Phosphate-buffered saline (PBS, 1×) was used for DNA dilution and hybridization. Before the recording of Raman spectra, 2 μL of the solution was added dropwise on the SERS substrate, and the measurement was performed after the solution dried naturally.

### Epitaxial Growth of the InGaN QWs

The QW structure was grown on a *c*-plane sapphire by metal–organic chemical vapor deposition (MOCVD, AIXTRON 200/4 RF). Precursors for the growth include ammonia (NH_3_), trimethylgallium (for the 2 μm n-type GaN base), triethylgallium (for QWs), and trimethylindium. Hydrogen and nitrogen were employed as the carrier gases for n-type GaN and QWs, respectively. The growth pressure was fixed at 200 mbar, and the growth temperature was varied in the range of 550–1120 °C. For the QW-free sample, the growth was stopped at the 2 μm n-type GaN base. To decorate the SERS substrates with Al nanoparticles, a 30 nm Al layer was deposited (0.5 Å/s) on the nitride surface using an e-beam evaporator (ULVAC, operation pressure: 3 × 10^–6^ Torr), followed by the rapid thermal annealing at 300 °C for 3 min in a N_2_ atmosphere.

### Spectrum Acquisition

Raman and photoluminescence (PL) spectra were excited by a 488 nm single-longitudinal-mode solid-state laser (Integrated Optics). For Raman spectra, a beam expander and a neutral density filter (Thorlabs) were used to control the beam size and power of the excitation light. The laser beam was focused by an objective (100× LMPlanFl, Olympus), with a numerical aperture of 0.8, mounted on an optical microscope (CX41, Olympus). After passing a dichroic mirror (Di01-R488-25x36, Semrock) and an edge filter (BLP01-488R-25, Semrock), SERS signals were detected by a spectrometer (Shamrock 500i, Andor). The laser power, spot size, and exposure time for each Raman spectrum were 18 mW, 700 nm, and 0.5 s, respectively. For SERS images, details on the setup and single-pixel algorithm can be found in the Supporting Information.

## Results and Discussion

**Figure 1:**
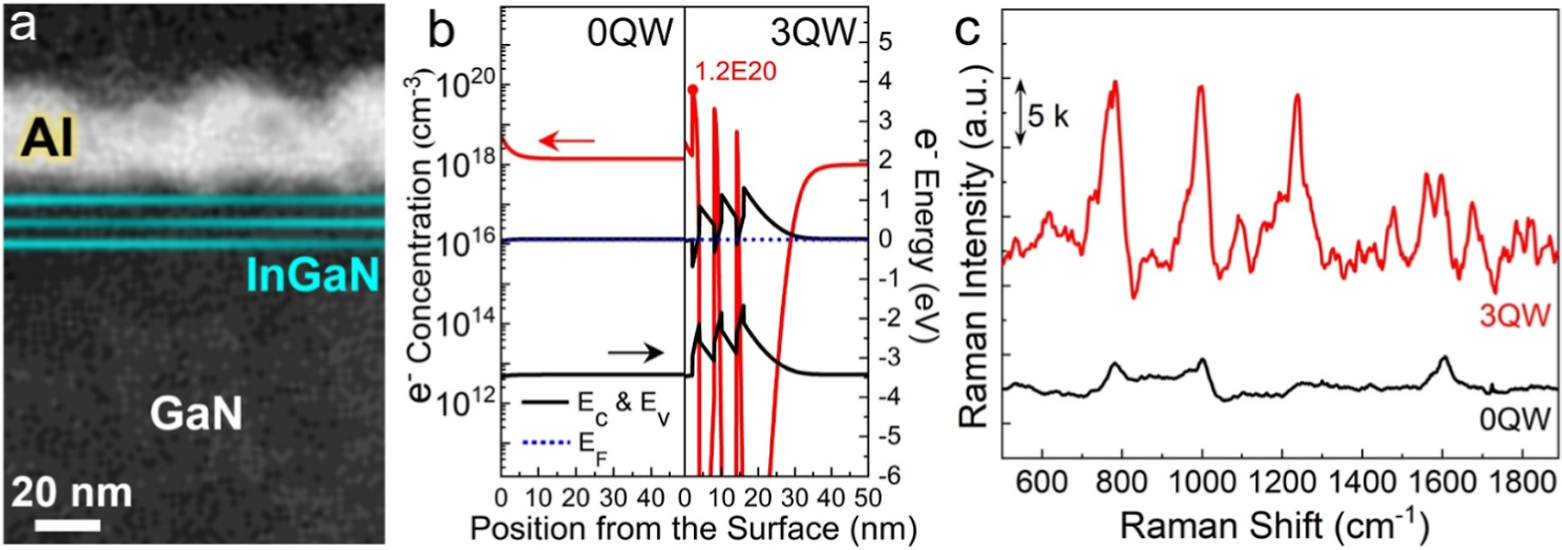
(a) Layer structure of the SERS substrate with InGaN quantum wells (QWs) and nanostructured Al surface. (b) Equilibrium band diagrams of the SERS structures with no QW (0QW, i.e., pure GaN) and 3-repeat InGaN/GaN QWs (3QW). The diagrams were simulated with the ohmic contact of Al/GaN. E_c_, E_v_, and E_F_ are the conduction band edge, valence band edge, and Fermi level, respectively. Electrons confined in the QW can reach a concentration as high as 1.2 × 10^20^ cm^–3^. (c) SERS spectra of the probe of KRAS ctDNA recorded on the 0QW and 3QW samples, showing enhanced Raman intensities on 3QW.

Figure 1a shows the layer structure of the QW for SERS detection. Characterization results by PL, scanning electron microscopy, and transmission electron microscopy are provided in Figure S1 in the Supporting Information. The key contribution from QWs to SERS is the abundant subsurface electrons,^12^ as revealed by the band diagrams in Figure 1b. The diagrams compare band bending and electron concentration of the substrates with no QW (0QW) and three-repeat InGaN/GaN QWs (3QW), which were simulated by solving self-consistent Poisson and drift-diffusion equations with the ohmic contact of Al.^16^ For the 0QW sample, the growth was stopped at the 2 μm n-type GaN base (Figure S1a in the Supporting Information). It is clear that the QWs provide many more electrons, whose position from the surface can be easily and precisely controlled by MOCVD.^12^ Raman enhancement on the 3QW is evidenced by Figure 1c, showing SERS signals of the probe KRAS ctDNA recorded on the surfaces with 0QW and 3QW. The intensified Raman peaks on 3QW are attributed to the two mechanisms responsible for SERS, i.e., the charge-transfer resonance and the localized surface plasmon resonance (LSPR).^12−14^ For charge-transfer resonance, the subsurface electrons trapped in QWs can be pumped (by laser) to the Al surface by tunneling through the 1.6 nm GaN cap layer (Figure S1a in the Supporting Information) and join the collective vibration for Raman scattering.^12,13^ Electrons transferred to Al can particularly intensify certain Raman modes owing to the vibronic selection rule,^17^ building up the selectivity of SERS sensing. For LSPR, which accounts for the major signal enhancement in SERS,^6,12^ it was found that the plasmonic coupling between the surface metal and QWs can be induced during the SERS measurement.^14^ In the metal-QW coupling, electrons in QWs are oscillating together with those on metal, making every metal nanosphere an intensity-boost hot spot and thus greatly increasing the number of SERS-active regions.^14^ The two enhanced resonances allow the QWs to form the “hot surface” by interconnecting the much densified hot spots. Detailed elucidation on the formation and effect of the SERS hot surface has been reported in our recent studies.^12−14^ On the 3QW surface, the much intensified signals shown in Figure 1c allow us to identify the presence of target DNA.

**Figure 2:**
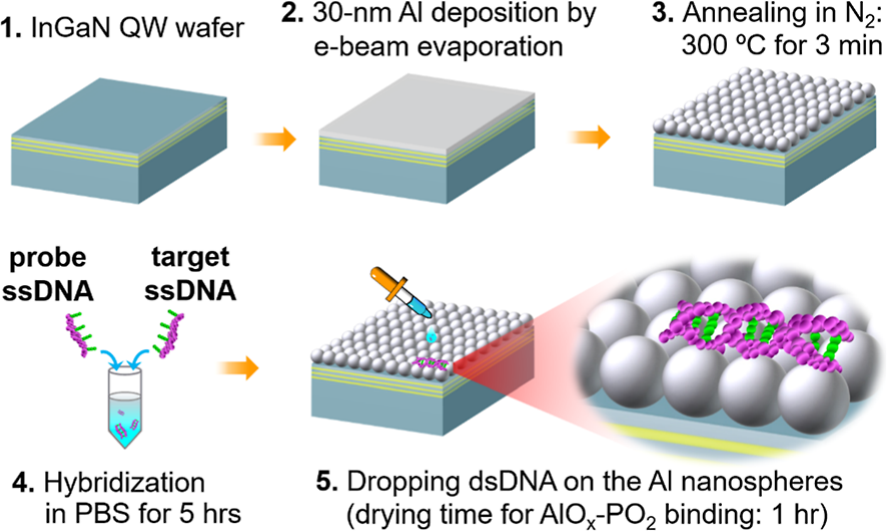
Schematic diagram showing the five steps for DNA detection by the SERS biochip with InGaN quantum wells (QWs). The hybridization of single-stranded DNA (ssDNA) in a phosphate-buffered saline (PBS) solution and self-immobilization of double-stranded DNA (dsDNA) on Al nanospheres expedite the preparation process and improve sensing performances. Native oxide formed on the Al surface can naturally bind the phosphate backbone of DNA. The 1 h AlO_*x*_-PO_2_ binding was carried out in the dry box at 25 °C and 50% humidity.

Figure 2 schematically describes the five steps to perform DNA detection on the QWs: (i) QW growth; (ii) Al deposition; (iii) annealing; (iv) hybridization; (v) dropping and detection. These steps have two unique features: (a) thiol-free adsorption of double-stranded DNA (dsDNA) on the Al nanospheres; (b) high-efficiency hybridization in the phosphate-buffered saline (PBS) solution. In comparison with conventional nucleic acid sensors,^2,3^ the two features simplify sample preparation and yield superior sensitivity. The Al surface texture was created by a 3 min annealing at 300 °C in N_2_, rendering a sphere-like morphology in the nanoscale (see Figure S1d in the Supporting Information) and reducing the undesired plasmon damping, both of which lead to enhanced LSPR for SERS.^18^ In addition, annealing the Al layer also expedites the formation of a thin and stable AlO_*x*_ layer on the Al nanospheres (see Figure S1e,f in the Supporting Information).^19^ It has been reported that the oxidized surface can provide favored binding sites for the phosphate diester backbone of DNA through an AlO_*x*_-PO_2_ interaction,^15,20^ which is not available on other commonly used metals (e.g., Au). As shown in the schematic, the AlO_*x*_-shelled nanospheres can support the full DNA strand by anchoring the phosphate backbone, which takes place during the 1 h wait in a dry box. This scheme skips the tedious processes of surface functionalization (usually with thiol linkers) and immobilization of aptamers,^21,22^ whose application potential was compromised by the high cost in labor and time, as well as by the risk of analyte contamination and information loss.^23^

More importantly, immobilizing DNA by AlO_*x*_-PO_2_ binding can unlock the full potential of SERS, i.e., exposing the entire DNA to the hot surface. In the conventional DNA assay by SERS, the hybridization is carried out by dropwise adding the solution of target nucleic acids on the probe, which is vertically immobilized on the sensing surface.^21^ Raman signals (specific to the hybridization process) are then detected at the hot spots of SERS. However, since the extraordinary signal boosting of SERS is only effective within 5 nm (∼9 nucleotides) from the surface (owing to the nature of charge transfer and LSPR of hot spots),^21,24,25^ most of the target nucleotides (far above the surface) are not detectable, unless the hairpin conformation or Raman reporter molecules are incorporated (with demanding procedures) in the DNA structure.^3,26,27^ Such a curb on SERS efficacy can be removed if the hybridized DNA is horizontally adsorbed on the Al nanospheres. As all of the nucleotides are exposed to the hot surface, minor changes in the nucleobases (due to hybridization or mutation) are more likely to be identified via spectral variance.

Another benefit of this simplified process comes from the 5 h hybridization in the PBS solution, which is expected to improve the sensitivity of DNA detection. The hybridization was carried out with the probe single-stranded DNA (ssDNA) concentration fixed at 1 × 10^–3^ M and the target ssDNA concentration diluted from 1 × 10^–3^ to 1 × 10^–13^ M. Cations in the PBS were utilized to prevent the repulsion between the negatively charged phosphates of DNA strands.^28^ In this way, the complementary ssDNA can freely move and twist in the liquid, leading to a multifold enhancement of the hybridization efficiency.^29^ Zippering the duplex DNA in PBS also allows us to increase the probe concentration (to catch more targets) without worrying about the “probe overcrowding” issue, which is often encountered on the solid metal surface.^30,31^ The problem is caused by strong electrostatic interaction between densely immobilized probes, resulting in suppressed affinity for targets and thus hindering the hybridization. After the hybridization in PBS, the dsDNA (2 μL) is drop-casted on the Al nanospheres. Compared with the vertically linked dsDNA that partially vibrates at the unlinked (upper) end, the dsDNA (with rigid duplex geometry) fully attached on the SERS chip is expected to produce a stabilized signal because of the reduced vibration.^26,27^

**Figure 3:**
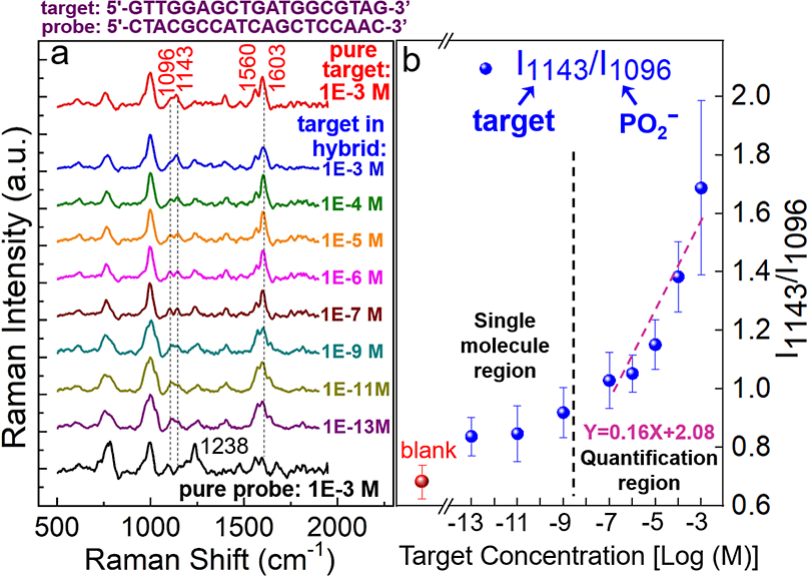
Sensitivity of the nitride SERS biochip. (a) SERS spectra of 19-mer DNA hybridized in PBS liquid with a fixed probe concentration (1 × 10^–3^ M) and decreased target ones (from 1 × 10^–3^ to 1 × 10^–13^ M). The spectra of single-stranded pure probe (1 × 10^–3^ M) and pure target (1 × 10^–3^ M) are also presented at the bottom and the top, respectively. Sequences of the probe and the target are listed above the spectra. In each spectrum, peak intensities are normalized to the one at 1000 cm^–1^. (b) Correlation between the intensity ratio *I*_1143_/*I*_1096_ and the target DNA concentration on a semilogarithmic scale. Error bars are the standard deviations of 10 spectra at each concentration. The less changed *I*_1143_/*I*_1096_ ratios at the concentrations below 1 × 10^–9^ M indicate that only few target DNAs are captured by the probe.

Figure 3a shows the Raman spectra of hybridized DNA on the SERS chip with 3QW. The spectra were recorded with the probe concentration fixed at 1 × 10^–3^ M and the target decreasing from 1 × 10^–3^ to 1 × 10^–13^ M. Each spectrum in the figure is an average of 10 measurements performed at different spots on the chip surface, and only the target signals with intensities at least 3 times the noise level (average intensity in the blank range at 500–550 cm^–1^) were recorded. The target DNA is of the sequence from KRAS (G12D mutation), which is responsible for over 40% of the patients with pancreatic ductal adenocarcinoma.^10^ The 1000 cm^–1^ peak corresponds to the breathing mode of pyridine rings in adenine,^31^ according to the SERS signals recorded with the four pure nucleobases provided in Figure S2a in the Supporting Information, i.e., adenine (A), cytosine (C), guanine (G), and thymine (T). Comparing the two ssDNA spectra (at the bottom and the top), one can see that the probe (C-rich) and target (G-rich) nucleobases are featured by the peaks at 1238 cm^–1^ and 1143 cm^–1^, respectively. Based on the references (Figure S2a in the Supporting Information), the probe feature (1238 cm^–1^) stems from the contribution of C and A bases, and the target one (1143 cm^–1^) is from the twisted mode involving G, A, and the PO_2_ backbone. Hybridization between the probe and target results in the concurrence of the two distinctive peaks (Figure S2b in the Supporting Information). Moreover, the Raman mode at around 1096 cm^–1^, corresponding to the PO_2_ phosphate backbone,^15^ is observable with all spectra in Figure 3a. The result suggests that the DNAs, whether in single- or double-stranded configuration, are preferentially tethered on the AlO_*x*_ surface in the horizontal orientation, echoing the observation reported by the Halas group.^15^ Further, as the concentration of target DNA decreases from 1 × 10^–3^ to 1 × 10^–13^ M, a clear peak shifting from 1143 cm^–1^ to 1096 cm^–1^ is observed. In other words, the signature of the target at 1143 cm^–1^ is more pronounced at high target concentrations, while the one of the PO_2_ backbone at 1096 cm^–1^ is more pronounced at low target concentrations. The gradual peak shifting can be quantified by plotting the intensity (*I*) ratio of *I*_1143_/*I*_1096_ as a function of the target concentration, as presented in Figure 3b. It is found that *I*_1143_/*I*_1096_ shows a clear concentration dependence on the target diluted from 1 × 10^–3^ M to 1 × 10^–7^ M, and the dependence is less noticeable at concentrations below 1 × 10^–7^ M. The two-step intensity-concentration dependence has been reported as an indication of single-molecule detection by SERS.^32^ In the range of 1 × 10^–3^–1 × 10^–7^ M (namely, the quantification region), the value of *I*_1143_/*I*_1096_ changes in correspondence with the analyte concentration, whose linear dependence in the semilogarithmic scale (with the regression equation shown in the figure) can be a prediction tool for the development of tumor. At concentrations below 1 × 10^–7^ M, although the three *I*_1143_/*I*_1096_ ratios are still larger than that of the negative control (i.e., *I*_1143_/*I*_1096_ of the pure probe), further reduction of the analyte quantity does not result in a clear change of signal intensity. The result implies that only few target DNAs are captured by the probe.^32^ Specifically, the number of targets within the laser spot (diameter: 700 nm) at the concentrations of 1 × 10^–9^ M, 1 × 10^–11^ M, and 1 × 10^–13^ M were estimated, respectively, to be 150, 1.5, and 0.015, based on the droplet area (diameter: 2 mm) on the SERS sample (see Figure S3 in the Supporting Information for details). These numbers agree with the single-molecule characteristic seen in Figure 3b. For other SERS biosensors, the single-molecule detection of DNA at 1 × 10^–13^ M was only achievable by labeling a Raman reporter,^3^ which is a time-consuming process. One should be reminded that differentiating the peak change at such a low analyte concentration without labeling is possible only when the hot-spot density is high enough to cover most of the nucleotides.

It is also worth noting that SERS signals at the single-molecule level typically exhibit increased intensity fluctuation,^32^ owing to the strong dependence of SERS enhancement factor on the distance between the analyte and the hot spot.^24,25^ This issue is not seen in our case, as demonstrated by the comparable error bars at concentrations from 1 × 10^–13^ to 1 × 10^–5^ M. The relatively large error bar at 1 × 10^–3^ M is caused by the aggregate of high-concentration DNA (shown in Figure S4 in the Supporting Information). The result suggests that signal reproducibility is improved by the hot surface on QWs (shown later). In addition to the peaks at 1143 cm^–1^ and 1096 cm^–1^, SERS intensities at 1603 cm^–1^ and 1560 cm^–1^ also exhibit a clear dependence on the target concentration. The particularly strong peak at 1603 cm^–1^ is suitable for SERS imaging, which is another vital tool for high-throughput screening. Table 1 summarizes the limit of detection (LOD) and process times of ctDNA detection by different SERS schemes. In our case, the LOD (2.1 × 10^–12^ M) was calculated with the equation: LOD = 3σ/slope, where σ is the standard deviation of *I*_1143_/*I*_1096_ obtained with 10 spots on the negative control (the sample with pure probes), and the slope is given by the fitted equation. 5 h hybridization was carried out at room temperature with a probe concentration of 1 × 10^–3^ M, and the time (5 h) was expected to be reduced when the temperature and the probe concentration were optimized.^33^ Our OW-based approach features the skipping of surface functionalization, fluorescent labeling, and probe immobilization without using any functional molecules other than the nucleic acid probe and target. This unique scheme not only expedites the detection process but also minimizes the risk of process contamination,^23^ allowing us to attain the pure information on the analyte in its native state.

**Table 1:** Process Time of Each Step for the Detection of ctDNA Sequences by Different SERS Schemes^a^

Group	ctDNA	substrate	surface functionalization	fluorescent labeling	probe decoration or immobilization	hybridization	LOD	total time
Zhou et al., 2016^2^	KRAS	Au + glass	54 h (T-rich ssDNA)	none	not specified	1 h	3 × 10^–16^ M	>55 h
Zhang et al., 2019^3^	KRAS	Ag + glass	8 h (3-mercaptopropyl-triethoxysilane in toluene)	4 h (Raman reporter, DSNB)	6.5 h (hairpin probe on Ag)	11.5 h	1.2 × 10^–16^ M	30 h
Kowalczyk et al., 2019^4^	BRAF	Au + GaN	1 h (6-mercaptohexan-1-ol)	none	3 h (probe thiolated on Au)	1 h	∼2.5 × 10^–11^ M (0.17 pg/μL)	5 h
this study	KRAS	Al + InGaN QWs	none	none	none	5 h	2.1 × 10^–12^ M	6 h

a The total time of this study includes the 1 h drying in the dry box.

**Figure 4:**
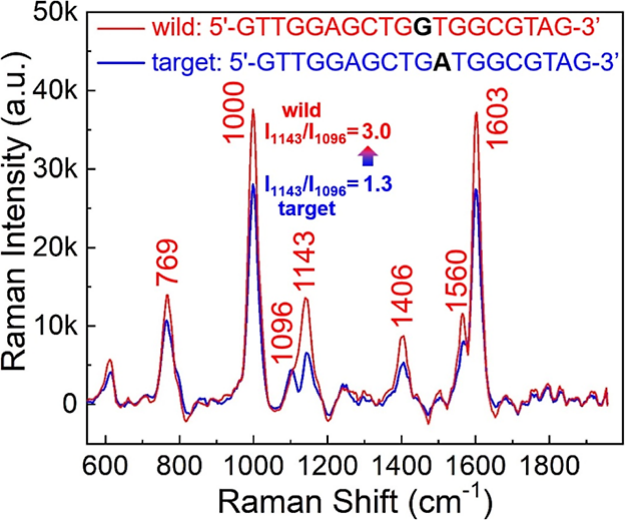
Selectivity of the nitride SERS biochip. SERS spectra of dsDNA hybridized with the probe (1 × 10^–3^ M) and (i) the perfectly matched target (1 × 10^–5^ M, in blue) and (ii) the single-base mismatched wild (1 × 10^–5^ M, in red). Sequences of the target and the wild DNAs are listed above the spectra. The peak enhancements at 769, 1000, 1143, 1406, 1560, and 1603 cm^–1^ are contributed mostly by the nucleobases, whose exposure to the SERS-active region is increased by the reduced hybridization efficiency due to the mismatched wild-type ssDNA.

Figure 4 compares the two Raman spectra of dsDNA hybridized by the probe (1 × 10^–3^ M) and (i) the perfectly complementary target (1 × 10^–5^ M) and (ii) the single-base mismatched wild-type ssDNA (1 × 10^–5^ M). Sequences of the target and wild samples are given above the spectra. Raw data of the figure are provided in Figure S5 in the Supporting Information. As indicated in the figure, it is found that replacing the A base with G leads to clear peak enhancement at 769, 1000, 1143, 1406, 1560, and 1603 cm^–1^, while the 1096 cm^–1^ peak from the PO_2_ backbone becomes less visible. The six enhanced peaks are believed to come from the nucleobases, whose exposure to the SERS hot surface is increased. This result can be understood by a fact: the nucleobases in dsDNA are more likely to be detected when they are more exposed to the SERS-active regions. Since the hybridization efficiency between the probe and the wild ssDNA is decreased by the single-base mismatch, nucleobases of the incompletely zipped DNA duplex are more likely to be exposed to the hot surface, thus producing increased base signals (compared to the case of a perfectly matched pair). In other words, when the nucleobases are fully wrapped within the two complementary ssDNA, the base signal (*I*_1143_) is less detectable than the outer phosphate backbone signal (*I*_1096_). As a result, *I*_1143_/*I*_1096_ (=1.3) of the matched hybrid (less exposed nucleobases) is lower than that (=3.0) of the mismatched hybrid (more exposed nucleobases). This distinction is readily observed without spectra subtraction, which is often adopted in the literature to highlight the subtle difference between the two spectra with a single-base mismatch.^34−36^ Our result shows that horizontally immobilizing the dsDNA on a hot surface can capture the full tumor genetic landscape.

**Figure 5:**
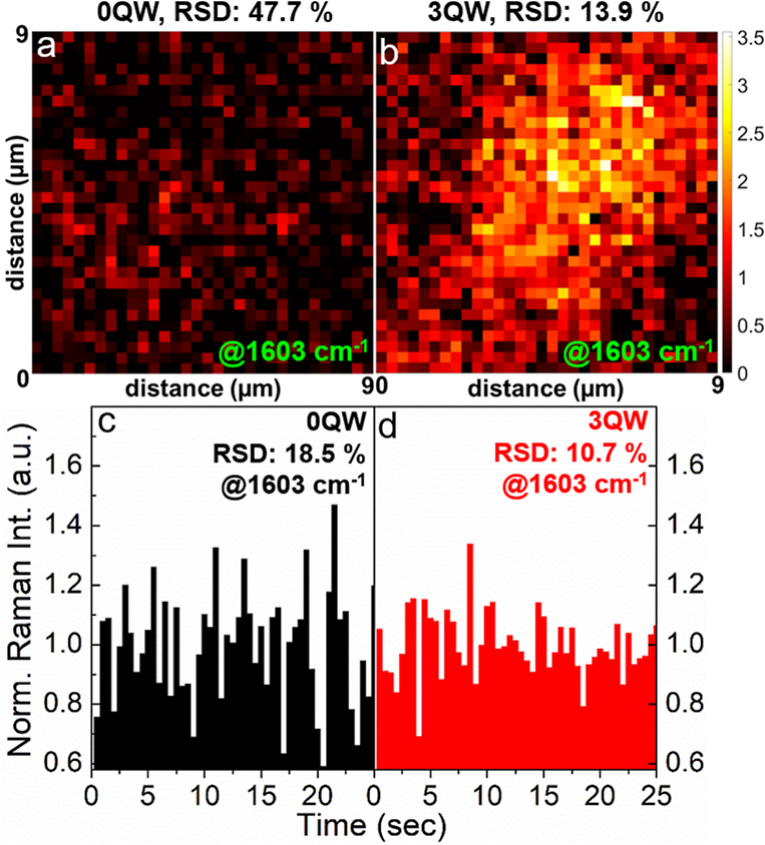
Stability of the nitride SERS biochip. 9 × 9 μm^2^ SERS images of the 1603 cm^–1^ peak from hybridized DNA (probe: 1 × 10^–3^ M; target: 1 × 10^–4^ M) recorded on the nitride chips with (a) no quantum wells (0QW); (b) 3-repeat QW (3QW). The images were attained by a beam-expansion optical setup, enabling the quick recording time of 3.5 min. Spatial uniformity is quantified by the relative standard deviation (RSD) of all pixel intensities. The time-dependent SERS intensities at a fixed position (with the same probe/target concentrations) on the surfaces of 0QW and 3QW are presented in (c) and (d), respectively. The displayed intensities in (c) and (d) are normalized to respective mean values on each sample. Compared to those on the 0QW surface, the reduced RSD values on 3QW indicate superior signal stability in space and time.

Figure 5a,b presents SERS images of the 1603 cm^–1^ peak, produced by the dsDNA (hybridized with 1 × 10^–3^ M probe and 1 × 10^–4^ M target) on the SERS chip with 0QW and 3QW, respectively. Unlike conventional SERS mappings,^13^ which take long hours to complete the point-by-point scanning at a tiny focus spot (∼1 μm^2^), the presented 9 × 9 μm^2^ images were captured with a single laser exposure with only 3.5 min. The wide-field images were attained by expanding the laser beam size. Since the excitation power density was reduced with the expanded laser beam, the target concentration was increased from 1 × 10^–5^ M for Figure 4 to 1 × 10^–4^ M for Figure 5, which allows us to see the difference in SERS signals on 0QW and 3QW. In the optical setup for SERS imaging (shown in Figure S6 in the Supporting Information), a digital micromirror device was used to generate a series of Hadamard patterns,^37^ which were enlarged and directed sequentially toward the DNA by the following lenses/mirrors to excite the SERS signals. The SERS images were reconstructed based on a single-pixel imaging algorithm (described in Figure S6 in the Supporting Information). SERS images obtained in this way can reach a spatial resolution of 276 × 276 nm^2^. The recording time can be further shortened by reducing the number of Hadamard patterns via the “compressive sampling” method.^37^ Such high-throughput SERS imaging with submicron resolution can provide real-time monitoring of tumor progression.^38^ Moreover, expanding the beam size without losing the speed/resolution of SERS imaging allows simultaneous collection of the spectra from multiple DNA samples, which is a powerful tool for rapid multiplex genetic testing.^39^ Comparing Figure 5a,b, it is clear that the one on 3QW exhibits a higher Raman intensity and larger signal area. Spatial uniformity was evaluated by the relative standard deviation (RSD) of peak intensity, reducing from 47.7% (0QW) to 13.9% (3QW). The improved Raman intensity and uniformity are ascribed to abundant oscillating charges on the QW surface, which promote charge-transfer resonance and LSPR.^12−14^

Not only improving the uniformity in space, but the QWs also enhance the uniformity in time, as demonstrated in Figure 5c,d. The figures display time-lapse analysis (detection duration: 25 s, without beam expansion) for the 1603 cm^–1^ peak on 0QW and 3QW. Similarly, the 3QW chip renders less intensity variation; i.e., the RSD decreases from 18.5% (0QW) to 10.7% (3QW). It is noted that the SERS intensities on the two samples show no declining trend over the 25 s, despite the relatively high excitation power (18 mW). The sustainable DNA signal can be attributed to the short exposure time (0.5 s) and high thermal conductivity (up to 177 W/m·K) of the GaN wafer,^40^ which provides efficient heat dissipation during the laser irradiation. The enhanced temporal stability of QWs is also due to the SERS-active hot surface, making the laser-heated DNA less likely to diffuse in and out of the Raman-boosting region.^13^ With the QWs, we were able to attain the wide-field image and time-lapse analysis at the target concentration down to the concentration of 1 × 10^–9^ M (see Figure S7 in the Supporting Information). RSDs at a very low concentration are still comparable to those in Figure 5b,d, supporting the presence of a hot surface and the full adsorption of duplex nucleotides on AlO_*x*_. The improved intensity stability in space and in time is strongly desired by SERS biosensors, whose development has been impeded for years by the severe signal fluctuation.^6^

## Conclusions

Our studies show that using Al nanospheres is an effective way to immobilize nucleic acids and to induce the SERS effect for DNA sensing. The single-molecule sensitivity and single-base selectivity of DNA detection are realized by the high-efficiency hybridization in PBS solution as well as the horizontal DNA orientation on the SERS surface. All of these are not possible without the hot surface formed on the InGaN QWs. In addition to the skipped processes of surface functionalization, fluorescent labeling, and probe immobilization, we further proposed a rapid SERS imaging technique to record a 9 × 9 μm^2^ intensity mapping in a 3.5 min single laser exposure. With simplified sample preparation and a rapid imaging tactic, this unique SERS biosensor can catch DNA quickly and accurately.

## Supporting Information Available

Additional experiment details; photograph of the full wafer; TEM/SEM images; SERS spectra of DNA; microscopic image showing the segregation of DNA; raw spectra of the single-base mismatch hybridization; optical setup; and SERS image of the target DNA at 1 × 10^–9^ M (PDF)
