# Supplementary material for: Single-Base Detection of DNA with Simplified Steps on InGaN Quantum Wells
Source: J Phys Chem B. 2025 Apr 29;129(18):4366–72. doi: 10.1021/acs.jpcb.5c00200 (PMC12067430; doi:10.1021/acs.jpcb.5c00200)
Supplement: Supplementary file 1 — jp5c00200_si_001.pdf [file jp5c00200_si_001.pdf]

Supporting Information

Single-Base Detection of DNA with Simplified Steps on InGaN Quantum Wells

Thi Anh Nguyet Nguyen, Ching-Lung Luo, Fan-Ching Chien, Kun-Yu Lai**

Department of Optics and Photonics, National Central University, Chung-Li, Taoyuan 32001, Taiwan.

E–mail : fcchien@dop.ncu.edu.tw; kylai@ncu.edu.tw

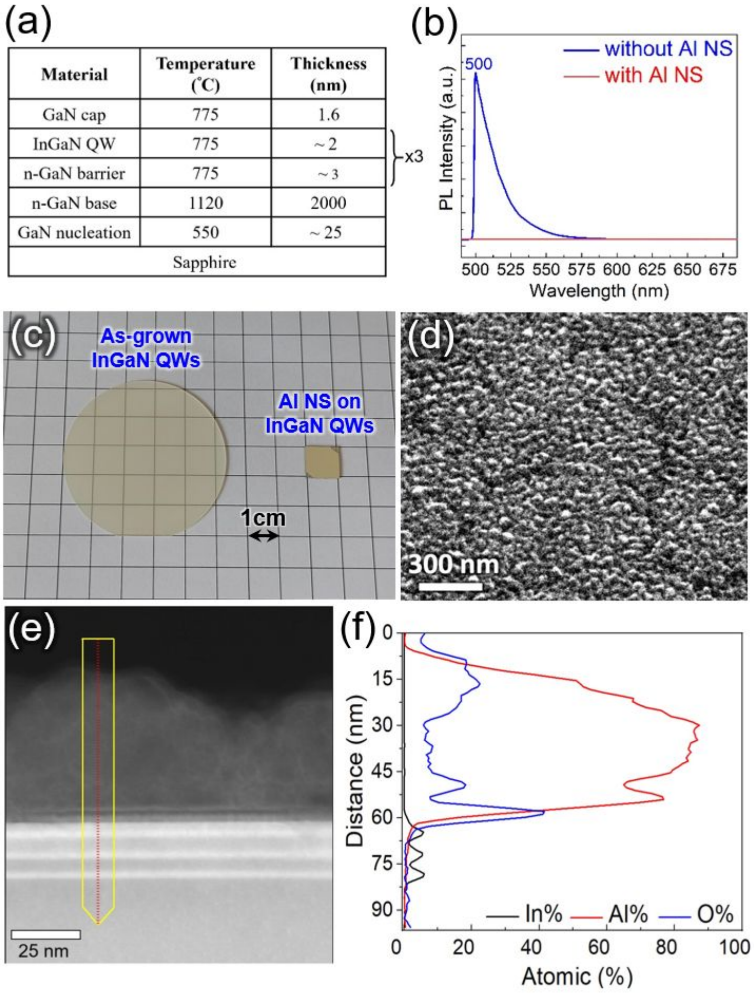

Material	Temperature (˚C)	Thickness (nm)
GaN cap	775	1.6
InGaN QW	775	~2
n-GaN barrier	775	~3
n-GaN base	1120	2000
GaN nucleation	550	~25
Sapphire		

Figure S1. Wafer-scale growth of the nitride wafer for SERS biochips. (a) Growth temperature and layer thickness of the QW-based SERS biochip for ctDNA sensing. (b) Photoluminescence (PL) spectra (excitation wavelength: 488 nm) of the InGaN QWs without and with Al nanospheres (NS). The peak at 500 nm on the bare QWs is suppressed by Al NS. (c) Photograph of the InGaN QW epilayer grown on a 2-inch sapphire substrate and a 1x1 cm^2 SERS chip, cleaved from the 2-inch wafer and decorated with Al NS. (d) Scanning electron microscopy (SEM) image of the Al NS fabricated on the QW epilayer. (e) Transmission electron microscopy (TEM) image and (f) the corresponding line scan along the red line in the TEM image, showing the atomic percentages of In, Al and O obtained by energy dispersive

spectroscopy (EDS). The increased O% on the Al surface indicates the formation of AlO_x. The oxygen at the Al/GaN interface is due to the native oxide on GaN.[1]

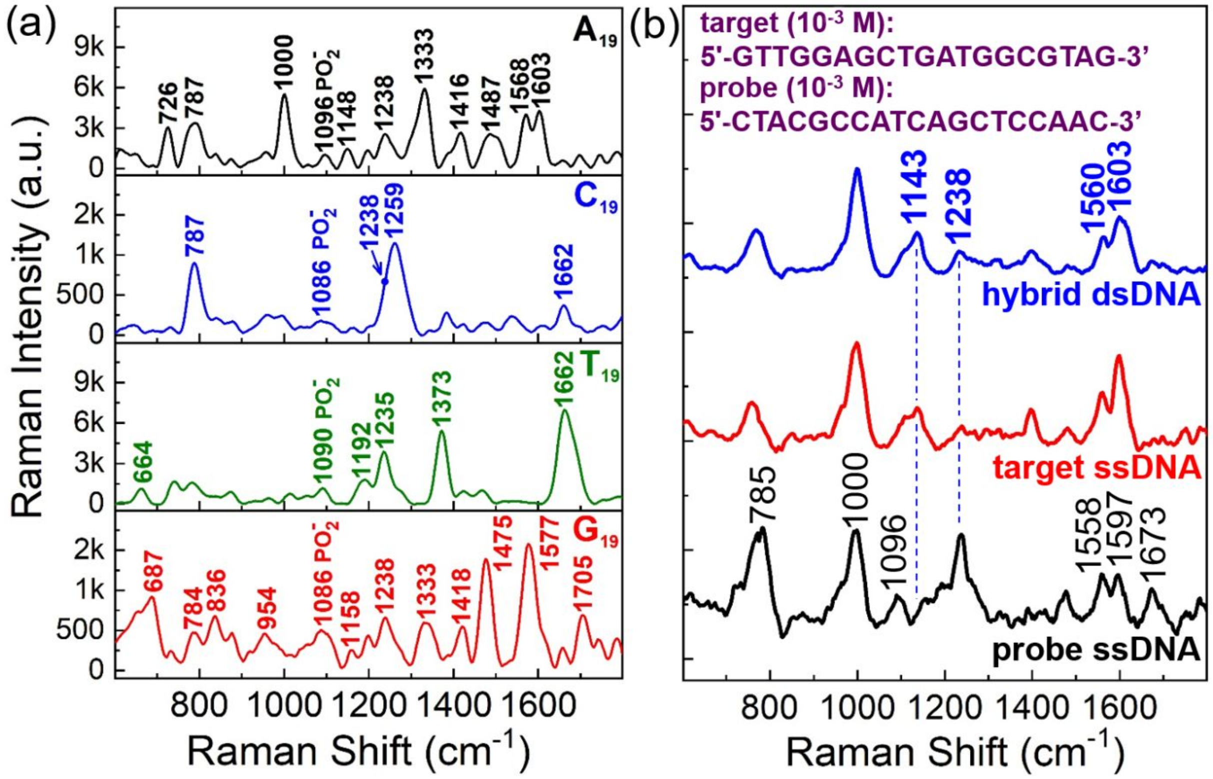

Figure S2. SERS signals of the four pure nucleobases and the hybridization event. (a) SERS spectra of the 19-mer nucleobases (A_{19}, C_{19}, T_{19}, and G_{19}, all at 1×10^{-3} M) recorded on the 3QW biochip. The displayed Raman characteristics of the four nucleobases are similar to those reported by other groups.[2,3] The vibration mode at 1086 - 1096 cm^{-1}, corresponding to the phosphate backbone,[3,4] are observed with all spectra. Note that the intensities from A_{19} and T_{19} (see the vertical scale) are stronger than those from C_{19} and G_{19}, and should contribute more to the feature peaks of ctDNA before and after hybridization. (b) SERS spectra (average of 10 measurements) of the probe (1×10^{-3} M) and the target (1×10^{-3} M) single-stranded DNA (ssDNA), and the corresponding double-stranded DNA (dsDNA) after hybridization recorded on the 3QW biochip. The distinct peak intensities at 1143, 1238, 1560 and 1603 cm^{-1} are used to identify the hybridization behavior. The slight difference in peak-position between the pure

bases in (a) and the ctDNA in (b) is attributed to the shift in the vibration modes when the base sequence is altered. Similar observation was also reported by other groups.[5-7]

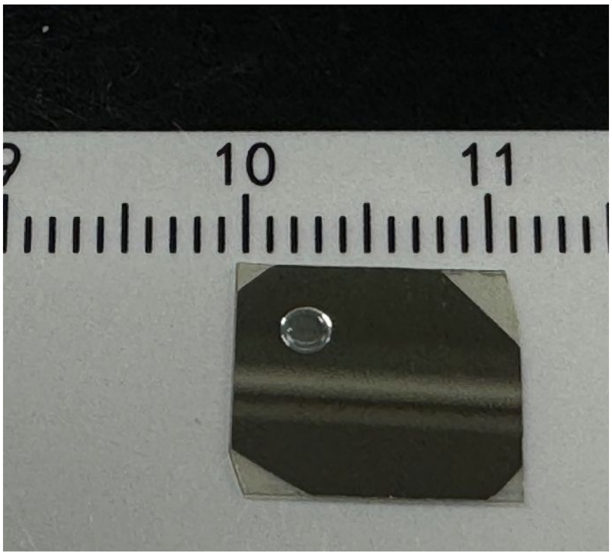

Figure S3. The area of a DNA droplet. The photograph of a DNA solution (volume: 2 μL) dropcasted on the SERS biochip, showing the diameter of 2 mm. Since the numbers of target ssDNA's at the concentrations of 1×10^{-9} M, 1×10^{-11} M and 1×10^{-13} M are 6.02×10^{14}, 6.02×10^{12} and 6.02×10^{10}, respectively, the numbers of target ssDNA's within the laser spot during the measurment can be estimated by the following calculations:

At the concentration of 1×10^{-13} M, the volume of a single DNA droplet: 2 μL

⇨ The number of DNA in a single droplet: $6.02\times10^{10}\times2\times10^{-6} = 1.2\times10^{5}$ copies

The area of a DNA droplet on the SERS biochip, as shown below in Fig. S3:

$\mathbf{A_{droplet}} = 1\times1\times\pi = 3.14\ mm^2 = 3.14\times10^{12}\ nm^2$

Assuming the DNA's are evenly distributed within the droplet area,

⇨ DNA density on the biochip:

$\mathbf{D_{DNA}}$ = 1.2x10^5 copies / 3.14x10^{12} nm^2 = 3.82x10^{-8} copies/nm^2

As mentioned in the manuscript, the laser spot size (diameter) is 700 nm,

⇨ The area of the laser spot: $\mathbf{A_{laser}}$ = 350x350xπ = 3.85x10^5 nm^2

⇨ The number of DNA within the laser spot:

$\mathbf{N_{1E\text{-}13}}$ = $\mathbf{A_{laser}}$ x $\mathbf{D_{DNA}}$ = (3.85x10^5 nm^2) x (3.82x10^{-8} copies/nm^2)

= 0.015 copies

Accordingly, for the target concentrations of 1x10^{-11} M and 1x10^{-9} M,

$\mathbf{N_{1E\text{-}11}}$ = 1.5 and $\mathbf{N_{1E\text{-}9}}$ = 150.

As shown in Fig. 3b in the manuscript, the ratio of I_{1143}/I_{1096} displays a slower decrease with the target concentration from 1x10^{-9} M to 1x10^{-13} M, compared to the case at the concentrations from 1x10^{-3} M to 1x10^{-7} M. Since $\mathbf{N_{1E\text{-}9}}$ is still larger than 1, the I_{1143}/I_{1096} at 1x10^{-9} M is slightly higher than those at 1x10^{-11} M and 1x10^{-13} M.

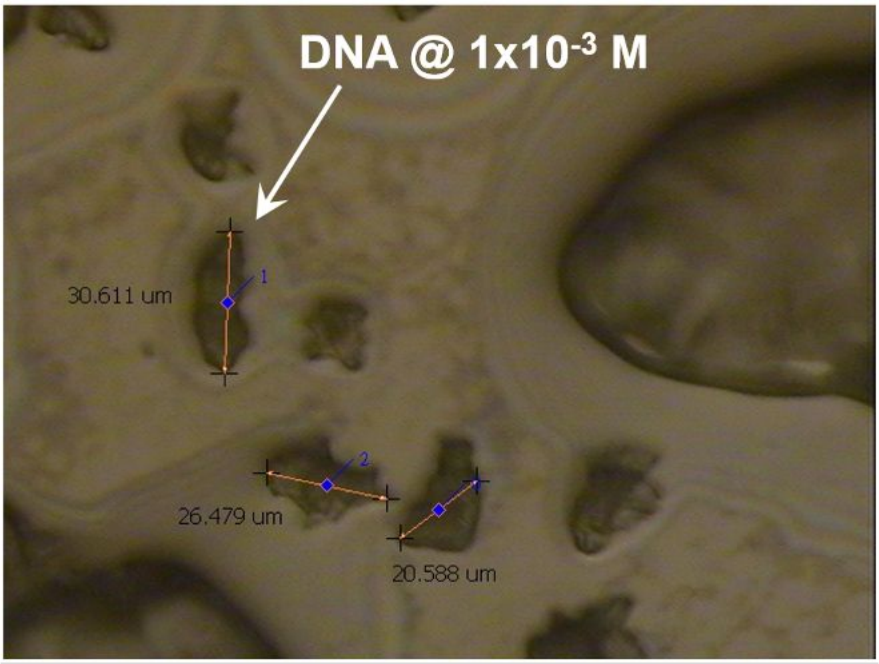

Figure S4. Optical image showing the aggregation of target DNA at the concentration of $1x10^{-3}$ M. The aggregation issue (less noticeable at lower concentrations) was the main contribution to the error bars in Fig. 3b in the main text.

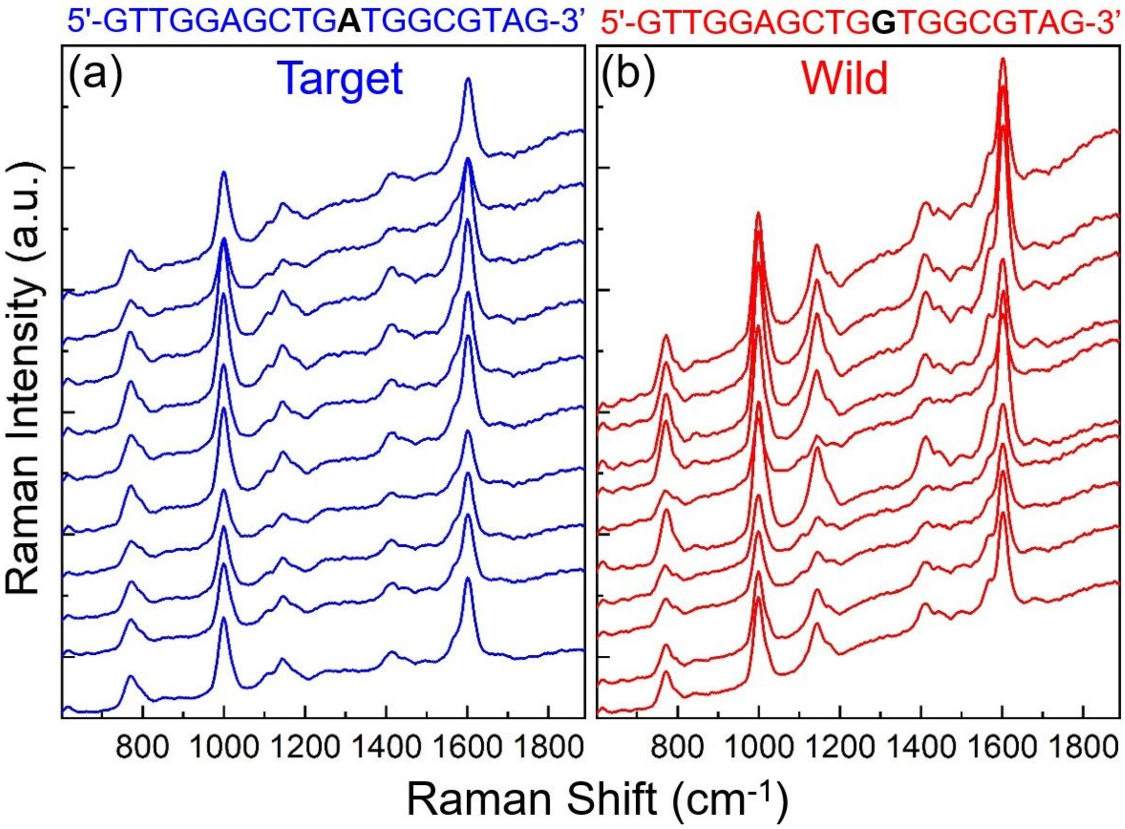

Figure S5. Raw spectra of Figure 4 in the main text. The ten SERS spectra of the dsDNA hybridized with the probe ctDNA (1×10^{-3} M) and: (a) the perfectly matched target (1×10^{-5} M); (b) the single-base mismatched wild (1×10^{-5} M). Sequences of the target and the wild ssDNA are listed above the spectra.

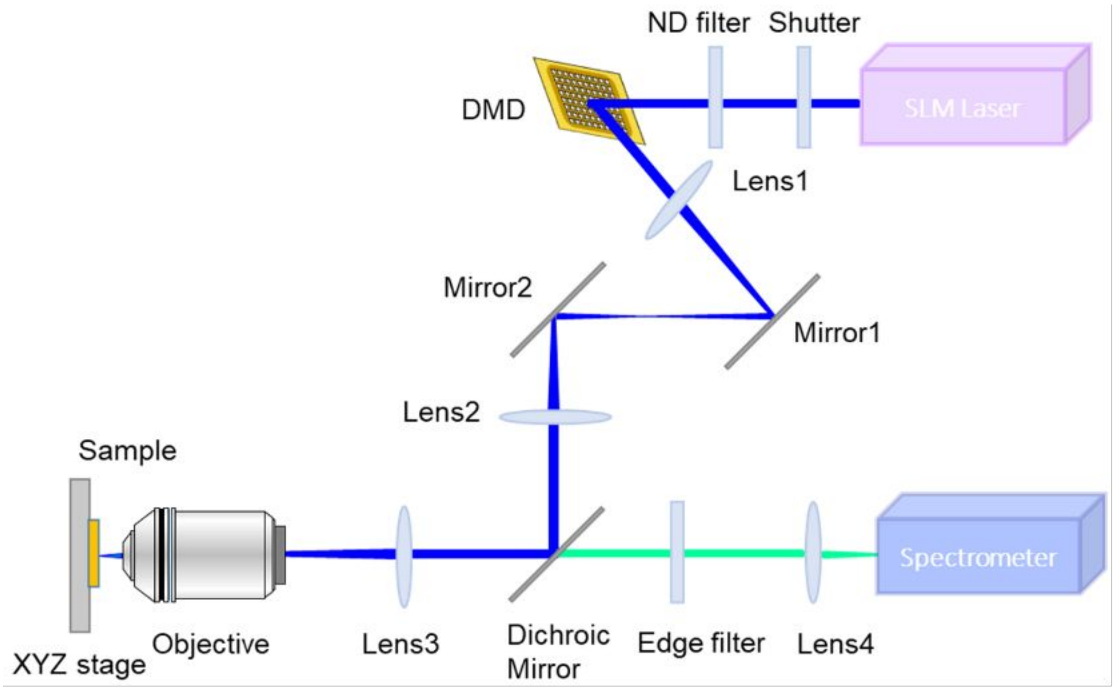

Figure S6. Optical setup for the high throughput SERS imaging of ctDNA, which was used to record the 3.5-min shots of the 1603-cm^{-1} signal presented in Figure 5(a) and 5(b) in the main text. To record the single-exposure SERS image, a 488-nm single-longitudinal-mode (SLM) laser was illuminated on a digital micromirror device (DMD, Texas Instruments) and its power was verified using a ND filter. The DMD acts as a modulator of reflective amplitude for arbitrary patterned illumination. After beam-size adjustment by the lens sets and the objective (100x LMPlanFl, Olympus), plane illumination with a series of designated Hadamard patterns was achieved to excite the SERS signals from a 9x9-μm^2 exposed area on the specimen. All SERS signals were filtered by a dichroic mirror and an edge filter, and then coupled into a spectrometer combined with an electron multiplying charge-coupled device (EMCCD) camera (Andor). Each detecting element of the camera chip was used to collect a series of SERS signals by the projected Hadamard patterns.[8] The SERS image for a specific range of wavenumber was reconstructed according to the single-pixel imaging approach as previously described,[8,9] which can be briefly expressed by

$$R(x,y) = \sum_{k=1}^{n^2} S_k \cdot h_k(x,y)$$

where $R(x,y)$ is the reconstructed image, $h_k(x,y)$ is the k'th Hadamard pattern, and S_k is the k'th SERS signal by the k'th Hadamard pattern illumination.[8] The n^2 Hadamard patterns are adopted to reconstruct the SERS image having a pixel number of $n \times n$. The pixel number of illumination pattern was 32×32 and the exposure time for each pattern illumination was 100 ms. Raman spectrum at a specific pixel of the SERS image can be obtained by assembling the intensities of the pixel in the reconstructed images of every wavenumber. In comparison with the conventional point-by-point mapping approach, SERS images captured in this way can be completed with a much shorter time.

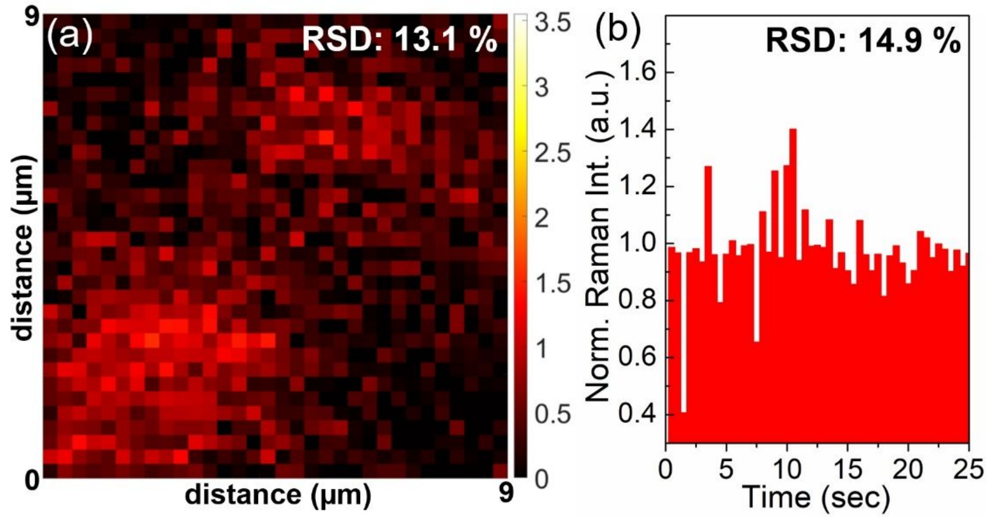

Figure S7. Spatial and temporal stability of the 1603-cm^{-1} SERS intensity at a low target DNA concentration (1x10^{-9} M). (a) 9×9 μm^2 image captured by the setup in figure S5. (b) 25-second time-dependent intensities (normalized to the mean value) at a fixed position. Similar to those presented in Figure 5 in the main text, these results were rendered by the hybridized DNA (probe: 1x10^{-3} M; target: 1x10^{-9} M) on the 3QW sample. The 1603-cm^{-1} signal at such low target concentration was not detectable on the 0QW sample.

References and notes:

[1] K. Prabhakaran, K. et al. Nature of Native Oxide on GaN Surface and Its Reaction with Al. *Appl. Phys. Lett.* **1996**, *69*, 3212—3214.

[2] Guerrini, L.; et al. Direct Surface-Enhanced Raman Scattering Analysis of DNA Duplexes. *Angew. Chem. Int. Ed. Engl.* **2015**, *54*, 1144—1148.

[3] Madzharova, F.; Heiner, Z.; Gühlke, M.; Kneipp, J. Surface-Enhanced Hyper-Raman Spectra of Adenine, Guanine, Cytosine, Thymine, and Uracel. *Phys. Chem. C* 2016, *120*, 15415—15423.

[4] Tian, S.; Neumann, O.; McClain, M. J.; Yang, X. ; Zhou, L.; Zhang, C.; Nordlander, P.; Halas, N. J. Aluminum Nanocrystals: A Sustainable Substrate for Quantitative SERS-Based DNA Detection, *Nano Lett.* **2017**, *17*, 5071—5077.

[5] Tang, H.-W.; Yang, X. B. ; Kirkham, J.; Smith, D. A. A characterization of four B16 murine melanoma cell sublines molecular fingerprint and proliferation behavior. *Anal. Chem.* **2007**, *79*, 3646—3653.

[6] Green, M. ; Liu, F.-M. ; Cohen, L. ; Kollensperger, P. ; Cass, T. Development of a novel wrinkle-structure based SERS substrate for drug detection applications. *Faraday Discuss.* **2006**, *132*, 269—280.

[7] Sapers, H. M. ; Hollis, J. R. ; Bhartia, R. ; Beegle, L. W. ; Orphan, V. J.; Amend, J. P. The Cell and the Sum of Its Parts: Patterns of Complexity in Biosignatures as Revealed by Deep UV Raman Spectroscopy. *Front. Microbiol.* **2019**, *10*, 679.

[8] Zheng, Z.; Wang, X.; Zheng, G.; Zhong, J. Hadamard Single-Pixel Imaging Versus Fourier Single-Pixel Imaging. *Optica.* **2017**, *25*, 19619−19639.

[9] Escobet-Montalbán, A.; Spesyvtsev, R.; Chen, M.; Saber, W. A. ; Andrews, M.; Herrington, C. S. ; Mazilu, M.; Dholakia, K. Wide-Field Multiphoton Imaging Through Scattering Media Without Correction. *Sci. Adv.* **2018**, 4, eaau1338.
